# An inflammatory biomarker‐based nomogram to predict prognosis of patients with nasopharyngeal carcinoma: an analysis of a prospective study

**DOI:** 10.1002/cam4.947

**Published:** 2016-11-10

**Authors:** Xiao‐Hui Li, Hui Chang, Bing‐Qing Xu, Ya‐Lan Tao, Jin Gao, Chen Chen, Chen Qu, Shu Zhou, Song‐Ran Liu, Xiao‐Hui Wang, Wen‐Wen Zhang, Xin Yang, Si‐Lang Zhou, Yun‐Fei Xia

**Affiliations:** ^1^ Department of Radiation OncologySun Yat‐sen University Cancer CenterState Key Laboratory of Oncology in South ChinaCollaborative Innovation Center of Cancer MedicineGuangzhouGuangdongChina; ^2^ Department of OncologyThe 421 Hospital of Chinese People’s Liberation ArmyGuangzhouGuangdongChina; ^3^ Department of Radiation OncologyAnhui Province Hospital Affiliated to Anhui Medical UniversityHefeiAnhuiChina

**Keywords:** Disease‐specific survival, inflammatory biomarkers, nasopharyngeal carcinoma, nomogram, prediction

## Abstract

Chronic inflammation plays an important role in tumor progression. The aim of this analysis was to evaluate whether inflammatory biomarkers such as the Glasgow prognostic score (GPS), the neutrophil‐lymphocyte ratio (NLR), the platelet‐lymphocyte ratio (PLR), and the lymphocyte‐monocyte ratio (LMR) could predict the prognosis of nasopharyngeal carcinoma (NPC). In this analysis, pretreatment GPS, NLR, PLR, LMR of 388 patients who were diagnosed as nonmetastatic NPC and recruited prospectively in the 863 Program No. 2006AA02Z4B4 were assessed. Of those, the 249 cases enrolled between December 27th 2006 and July 31st 2011 were defined as the development set. The rest 139 cases enrolled between August 1st 2011 and July 31st 2013 were defined as the validation set. The variables above were analyzed in the development set, together with age, gender, Karnofsky performance score, T stage, and N stage, with respect to their impact on the disease‐specific survival (DSS) through a univariate analysis. The candidate prognostic factors then underwent a multivariate analysis. A nomogram was established to predict the DSS, by involving the independent prognostic factors. Its predction capacity was evaluated through calculating Harrell's concordance index (C‐index) in the validation set. After multivariate analysis for the development set, age (≤50 vs. >50 years old), T stage (T1–2 vs. T3–4), N stage (N0–1 vs. N2–3) and pretreatment GPS (0 vs. 1–2), NLR (≤2.5 vs. >2.5), LMR (≤2.35 vs. >2.35) were independent prognostic factors of DSS (*P* values were 0.002, 0.008, <0.001, 0.004, 0.018, and 0.004, respectively). A nomogram was established by involving all the factors above. Its C‐index for predicting the DSS of the validation set was 0.734 (standard error 0.056). Pretreatment GPS, NLR, and LMR were independent prognostic factors of NPC. The nomogram based on them could be used to predict the DSS of NPC patients.

## Introduction

Nasopharyngeal carcinoma (NPC) is a distinct type of head and neck cancers because of its unique ethnic and geographic distributions, and pathological and clinical characteristics 1, 2, 3, 4. Currently, the gold standard for predicting the prognosis of NPC patients is the Union Internationale Contre le Cancer/American Joint Cancer Committee (UICC/AJCC) TNM staging system. However, there are mysterious heterogeneities of clinical outcomes in NPC patients with the same TNM stage 5, 6, 7, 8. The possible interpretation may be that the TNM staging system is merely based on the anatomy of tumor invasion and did not take into account the functional status of tumor cells or patient body. Therefore, more and more approaches have been made to explore the functional biomarkers which are able to improve prognosis prediction of NPC 9, 10.

Since the hypothesis of Virchow, there have been more and more evidences supporting that inflammation plays an important role in cancer progression 11, 12. Some inflammatory biomarkers, such as Glasgow prognostic score (GPS), neutrophil‐lymphocyte ratio (NLR), platelet‐lymphocyte ratio (PLR), and lymphocyte‐monocyte ratio (LMR), can be now easily tested and have been demonstrated to be related to the prognosis of various cancers 13, 14, 15, 16, 17, 18. These biomarkers may be used as practical predictors in the daily clinical work. However, few studies regarding these biomarkers in patients with NPC are available 19, 20, 21. The prognostic values of these biomarkers for NPC remain uncertain. And most of the studies focusing on inflammatory predictors, especially in NPC patients, analyzed only one of the biomarkers without considering the others. Moreover, these studies were almost retrospective.

The aim of this analysis was to investigate the prognostic role of the inflammatory biomarkers mentioned above (GPS, NLR, PLR, and LMR) in NPC, on the basis of data from a prospective study. And a nomogram was also developed, by involving the independent prognostic factors, to predict the survival of NPC patients.

## Patients and Methods

### Patient selection

The data used in this analysis was from patients enrolled between December 27th 2006 and July 31st 2013, in a prospective study which was referred to as ‘A Study of New Individualized Treatment Strategies for Nasopharyngeal Carcinoma Based on the biological behavior and molecular characteristics’ (the National High Technology Research and Development Program of China (863 Program) No. 2006AA02Z4B4). The sponsorship certificate of the Ministry of Science and Technology of China for the study was shown as Fig. S1.

Of those patients, the ones enrolled between December 27th 2006 and July 31st 2011 and completed 5‐year follow‐up were used for exploring the association between inflammatory biomarkers and prognosis of NPC, and developing a nomogram based on these markers (development set). The ones enrolled between August 1st 2011 and July 31st 2013 were used as an independent dataset for validation of the nomogram (validation set). The study was approved by the Ethics Committee of our hospital. And all patients signed informed consent before treatment.

Patients who were pathologically diagnosed as NPC in our hospital and initially treated by the corresponding author were consecutively involved in the study. All patients had detailed medical records, including magnetic resonance imaging of head and neck, whole‐body bone scan and thoraco‐abdominal computed tomography (or chest radiograph plus abdominal ultrasonography) for staging. Stage of the patients enrolled before January 1st 2010 was determined based on the 6th edition of UICC/AJCC TNM staging system. Stage of the ones enrolled after January 1st 2010 was determined based on the 7th edition. But for convenience, all the patients were restaged in this analysis according to the 7th edition 22, 23. We excluded patients from this analysis if they had any of these: (1) Karnofsky performance score (KPS) <70’; (2) distant metastases before or during radiotherapy (RT); (3) signs of infection before RT; (4) application of colony‐stimulating factors such as erythropoietin before RT; (5) RT uncompleted (≥1 fraction missing); (6) severe dysfunction of heart, lung, liver, or kidney and unsuitable for RT.

### Treatment

The treatment strategies for all the patients were based on National Comprehensive Cancer Network Guidelines. The cases with early‐stage (T1–2N0) diseases were treated with RT alone. The cases with locoregionally advanced‐stage diseases were treated with concurrent chemo‐radiotherapy.

The regimen of concurrent chemotherapy (CCT) was nedaplatin 80 mg/m^2^ d1 plus 5‐flurouracil 500 mg/m^2^ per day d2–5 every 3 weeks. A total of 2–3 cycles of CCT were applied during RT. If grade 3 to 4 hemopoietic, renal or hepatic disorder of Common Terminology Criteria for Adverse Events appeared, CCT was delayed until the disorder recovered to grade 1 or disappear, and the dose was decreased by 20% in the subsequent cycles. CCT was terminated if delay time lasted more than 2 weeks or twice appearance of any grade 4 adverse event appeared.

The target definition, delineation and dosage of RT were based on the standard of our hospital 24. Conventional 2‐dimensional RT consisted of two lateral opposing facio‐cervical fields to cover nasopharynx and the upper cervical lymphatic drainage region, and a lower anterior cervical field to cover the lower cervical region. After a dose of 36–40 Gy‐irradiated, two opposing lateral preauricular fields were used for the primary region, and anterior split neck fields were used for the cervical region instead. The primary tumor was given a total dose of 60–78 Gy, according to the tumor remission rate. In intensity‐modulated radiotherapy (IMRT), a total dose of 66–72 Gy was given to the gross tumor of nasopharynx, 60–70 Gy to the positive neck lymph nodes, 60 Gy to the high‐risk region, and 50–54 Gy to the prophylactic irradiation region.

### Follow‐up

After treatment, follow‐up was made by telephone, letter, or outpatient interview trimonthly for the first 3 years, semiannually for the fourth and fifth years and annually thereafter. All the patients were followed up until death from NPC or July 31st 2016, whichever came first. Causes of deaths were determined through death certificates, which were supplemented with medical records if necessary.

The primary endpoint of this analysis was the disease‐specific survival (DSS). It was defined in this analysis as the percentage of patients of a dataset who did not die from NPC in a defined period of time. The date to start calculating the DSS was defined as the date on which RT began.

### Assessment and definitions of inflammatory biomarkers

Complete blood count and routine biochemistry test, including C‐reactive protein (CRP) level and albumin (ALB), of each patient was applied 1 week before radiotherapy.

The GPS was defined as follows: (1) GPS 2’, simultaneous appearance of elevated CRP level above the upper normal range (CRP > 10 mg/L) and hypoalbuminemia (ALB <35 g/L); (2) GPS 1’, appearance of elevated CRP or hypoalbuminemia; (3) GPS 0’, CRP and serum albumin was within normal range 13.

The definition of NLR was the ratio of neutrophil count to lymphocyte count. PLR was the ratio of platelet count to lymphocyte count. And LMR was the ratio of lymphocyte count to monocyte count. Receiver operating characteristic (ROC) curves for predicting the 5‐year DSS were plotted to find out the optimum cut‐off values for the NLR, PLR, and LMR.

### Statistical analysis

In the development set, a chi‐square test was performed to assess the distribution balance of clinical characteristics between patients with different GPS, NLR, PLR, or LMR. Pearson correlation analyses were applied to analyze the correlation among NLR, PLR, and LMR. A univariate analysis through the Kaplan–Meier approach was then performed to examine the association of the 5‐year DSS with the clinical and inflammatory parameters, including gender (male vs. female), age (≤50 vs. >50 years old), KPS (≤80’ vs. >80’), clinical stage (I–II vs. III–IV), T stage (T1–2 vs. T3–4), N stage (N0–1 vs. N2–3), and pretreatment GPS (0’ vs. 1–2’), NLR, PLR, and LMR. The difference in the 5‐year DSS was assessed by the log‐rank test. Parameters considered as possible prognostic factors in the univariate analysis would went through a multivariable analysis, to check if they were independent prognostic factors. Hazard ratio (HR) and 95% confidence interval (CI) of the factors were also calculated. A nomogram enrolling independent prognostic factors was built to predict 1‐year, 3‐year, and 5‐year DSS of NPC patients through the Cox regression model, just as Liu et al. did in development of a nomogram for predicting prognosis of esophageal carcinoma 25.

Finally, the predictive accuracy on DSS was validated by calculating the Harrell's concordance index (C‐index) of the nomogram in the validation set. Because these patients just completed a 3‐year follow‐up, validation was performed for the prediction of the 3‐year DSS. And a calibration plot was also depicted for the 3‐year DSS.

**Figure 1:**
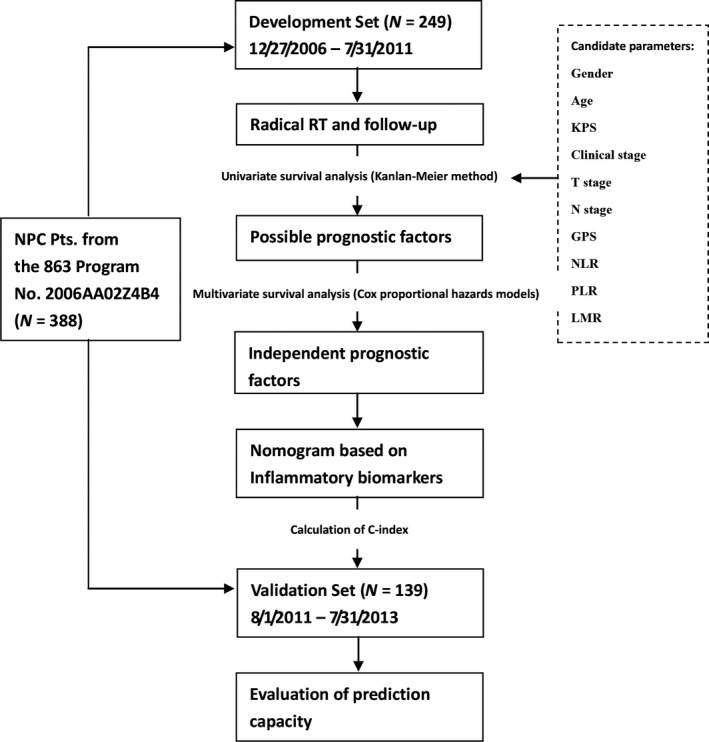
The entire procedure of this analysis. A total of 388 patients from the 863 Program No. 2006AA02Z4B4 were eligible for this analysis: 249 cases with complete 5‐year follow‐up were allocated to the development set, and the rest 139 cases were allocated to the validation set. Data of the GPS, the NLR, the PLR, the LMR and other characteristics (KPS) were assessed for the patients of the development set. Possible prognostic factors were picked out by the univariate survival analysis. The multivariate survival analysis was then performed to screen out the independent prognostic factors. A nomogram enrolling independent prognostic factors was developed. Finally, its predictive accuracy was validated through calculation of its Harrell's concordance index (C‐index) on 3‐year disease‐specific survival in the validation set. GPS, Glasgow prognostic score; LMR, lymphocyte‐monocyte ratio; NLR, neutrophil‐lymphocyte ratio; PLR, platelet‐lymphocyte ratio; KPS, Karnofsky performance score.

The statistical analysis was made by IBM SPSS Statistics 16.0 (IBM Co., Armonk, New York, US) and R 3.2.4 (R Foundation, Vienna, Austria). A two‐sided *P* < 0.05 was considered to indicate statistical significance. The entire procedure of this analysis was summarized in Figure 1.

## Results

### Cutoff values of inflammatory biomarkers

Through the ROC curves, the optimum cut‐off values of NLR, PLR, and LMR for the 5‐year DSS were 2.5, 166, and 2.35, respectively (Fig. S2). The areas under curve (AUC) for NLR, PLR, and LMR were 0.618 (*P* < 0.001), 0.607 (*P* = 0.008), and 0.667 (*P* < 0.001), respectively.

### Baseline clinical and characteristics

Finally, a total of 388 patients were eligible for this analysis: 249 cases in the development set and 139 cases in the validation set. Comparison of the clinical characteristics in the development set between patients with different GPS, NLR, PLR, or LMR was shown in Table 1. For patients with high GPS, there were more cases with N2–3 disease (67.5% vs. 49.7%, *P *= 0.040), less cases with undifferentiated carcinoma (77.5% vs. 92.3%, *P *= 0.004), compared with those who had low GPS. For patients with high NLR, there were more cases with N2–3 disease (61.0% vs. 46.9%, *P *= 0.030), compared with those who had low NLR. And for patients with low LMR, there were more cases with Stage III–IV disease (94.7% vs. 83.8%, *P *= 0.018), more cases with T3–4 disease (89.4% vs. 67.0%, *P* < 0.001), and less cases with undifferentiated carcinoma (84.2% vs. 92.4%, *P* < 0.001), compared with those who had high LMR.

**Table 1:** Baseline clinical characteristics of the development set with different GPS, NLR, PLR or LMR

Characteristics	Cases	GPS		NLR		PLR		LMR	
		0’	1–2’	≤2.5	>2.5	≤166	>166	≤2.35	>2.35
Gender		*P *= 0.540		*P *= 0.394		*P *= 0.831		*P *= 0.793	
Male	184	156	28	113	71	142	42	57	127
Female	65	53	12	36	29	51	14	19	46
Age		*P *= 0.090		*P *= 0.611		*P *= 0.214		*P *= 0.551	
≤50	164	133	31	100	64	131	33	48	116
>50	85	76	9	49	36	62	23	28	57
KPS		*P *= 0.330		*P *= 0.922		*P *= 0.074		*P *= 0.612	
≤80	54	43	11	32	22	37	17	18	36
>80	195	166	29	117	78	156	39	58	137
Clinical stage		*P *= 0.556		*P *= 0.137		*P *= 0.716		*P *= 0.018	
I–II	32	28	4	23	9	24	8	4	28
III–IV	217	181	36	126	91	169	48	72	145
T stage		*P *= 0.862		*P *= 0.536		*P *= 0.576		*P* < 0.001	
T1–2	65	55	10	41	24	52	13	8	57
T3–4	184	154	30	108	76	141	43	68	116
N stage		*P *= 0.040		*P *= 0.030		*P *= 0.640		*P *= 0.779	
N0–1	118	105	13	79	39	93	25	35	83
N2–3	131	104	27	70	61	100	31	41	90
IMRT		*P *= 0.676		*P *= 0.320		*P *= 0.393		*P *= 0.773	
No	180	150	30	105	75	137	43	54	126
Yes	69	59	10	44	25	56	13	22	47
Undifferentiated carcinoma		*P *= 0.004		*P *= 0.082		*P *= 0.487		*P *= 0.045	
No	25	16	9	19	6	18	7	12	13
Yes	224	193	31	130	94	175	49	64	160
CCT		*P *= 0.945		*P *= 0.197		*P *= 0.463		*P *= 0.984	
No	13	11	2	10	3	9	4	4	9
Yes	236	198	38	139	97	184	52	72	164

KPS, Karnofsky performance score; IMRT, intensity‐modulated radiotherapy; CCT, concurrent chemotherapy; GPS, Glasgow prognostic score; LMR, lymphocyte‐monocyte ratio; NLR, neutrophil‐lymphocyte ratio; PLR, platelet‐lymphocyte ratio.

Results of Pearson correlation analyses were shown in Figure S3. There were moderate positive correlation between NLR and PLR (*r *= 0.58, *P* < 0.001). However, there were weak negative correlations between NLR and LMR (*r *= −0.340, *P* < 0.001), and between PLR and LMR (*r *= −0.213, *P *= 0.001).

### Survival analysis

**Figure 2:**
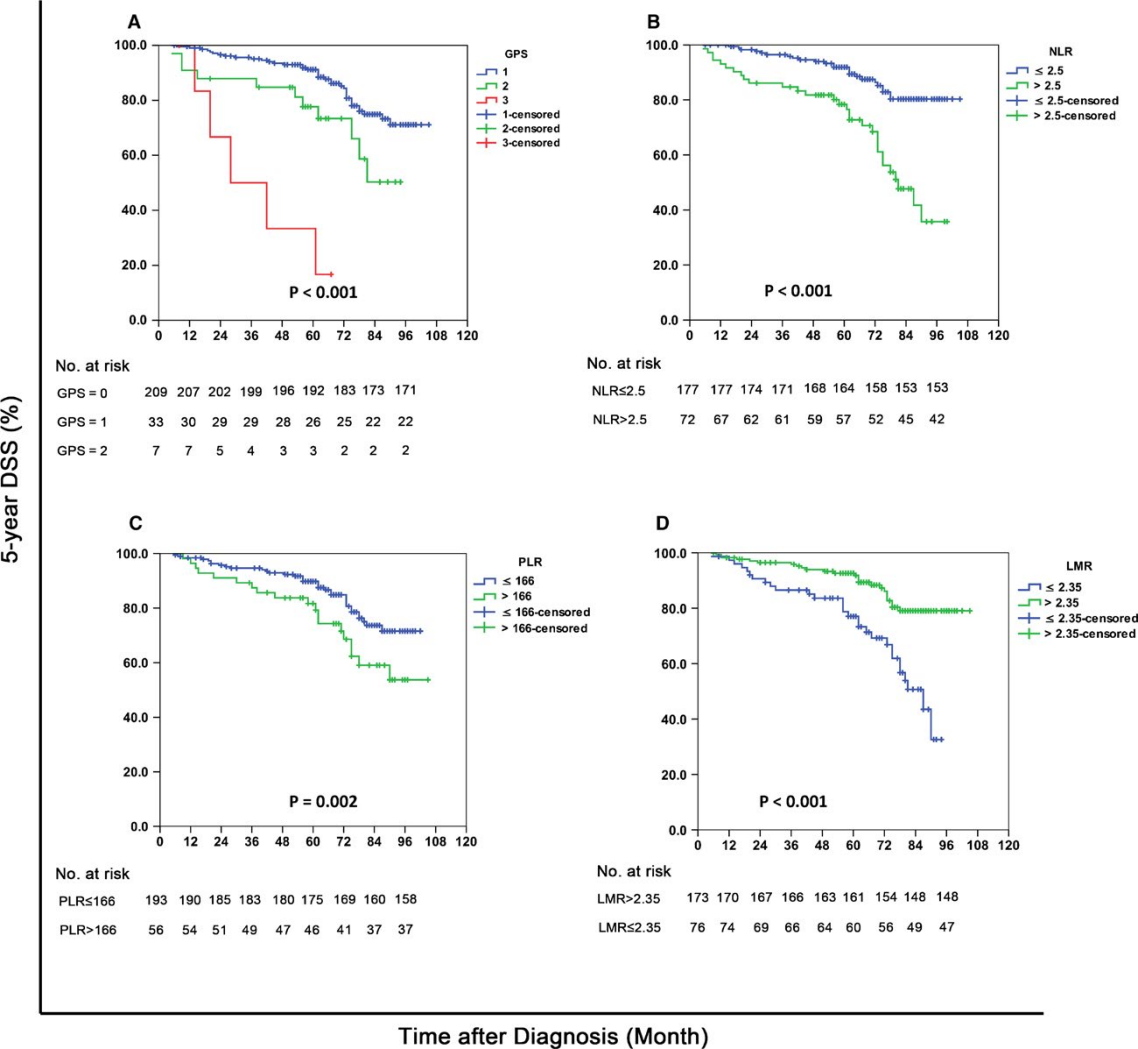
The 5‐year DSS curves of the development set, grouped by the GPS, the NLR, the PLR, the LMR. (Panel A): survival curves of patients with different GPS. (Panel B): survival curves of patients with different NLR. (Panel C): survival curves of patients with different PLR. (Panel D): survival curves of patients with different LMR. GPS* *= 1–2’, NLR > 2.5, PLR > 166 and LMR ≤ 2.35 were statistically associated with poor 5‐year DSS (*P* values were <0.001, <0.001, 0.002 and <0.001, respectively). DSS, disease‐specific survival; GPS, Glasgow prognostic score; LMR, lymphocyte‐monocyte ratio; NLR, neutrophil‐lymphocyte ratio; PLR, platelet‐lymphocyte ratio.

The results of the univariate and multivariate survival analysis for patients of the development set were shown in Table 2. Through the log‐rank test, age >50 years old, T3–4, N2–3, GPS* *= 1–2’, NLR > 2.5, PLR > 166 and LMR ≤ 2.35 were factors statistically associated with poor 5‐year DSS (*P* values were 0.035, 0.006, 0.005, <0.001, <0.001, 0.002 and <0.001, respectively). The survival curves of the patients grouped by GPS, NLR, PLR, and LMR were shown in Figure 2.

**Table 2:** Univariate and multivariate analysis on factors of the 5‐year DSS in the development set

Characteristics	5‐year DSS/%	Univariate analysis		Multivariate analysis	
		HR (95% CI)	*P* value	HR (95% CI)	*P* value
Gender			0.548		
Female	78.8	1.000			
Male	76.9	1.201 (0.662–2.178)			
Age			0.035		0.003
≤50	82.7	1.000		1.000	
>50	70.1	1.779 (1.043–3.035)		2.484 (1.364–4.522)	
KPS			0.280		
≤80	74.1	1.000			
>80	79.5	0.715 (0.389–1.315)			
T stage			0.006		0.008
T1–2	92.4	1.000		1.000	
T3–4	73.2	3.616 (1.440–9.077)		3.618 (1.402–9.339)	
N stage			0.005		0.028
N0–1	85.2	1.000		1.000	
N2–3	72.4	2.284 (1.282–4.067)		1.995 (1.079–3.689)	
GPS			<0.001		0.020
0	81.8	1.000		1.000	
1–2	60.0	2.999 (1.667–5.396)		2.362 (1.142–4.883)	
NLR			<0.001		0.049
≤2.5	86.4	1.000		1.000	
>2.5	58.3	3.438 (2.008–5.888)		1.939 (1.004–3.761)	
PLR			0.002		0.451
≤166	81.9	1.000		1.000	
>166	66.1	1.865 (1.067–3.262)		1.225 (0.695–2.269)	
LMR			<0.001		0.048
≤2.35	61.8	1.000		1.000	
>2.35	85.5	0.327 (0.191–0.559)		0.547 (0.298–0.996)	

HR, hazard ratio; CI, confidence interval; KPS, Karnofsky performance score; GPS, Glasgow prognostic score; NLR, neutrophil‐lymphocyte ratio; PLR, platelet‐lymphocyte ratio; LMR, lymphocyte‐monocyte ratio; DSS, disease‐specific survival.

The age, T stage, N stage, GPS, PLR, NLR, and LMR then went through multivariate analysis. Among these factors, age, T stage, N stage, GPS, NLR, and LMR maintained their prognostic significance in the multivariate analysis (*P* values were 0.003, 0.008, 0.028, 0.020, 0.049, and 0.048, respectively).

**Figure 3:**
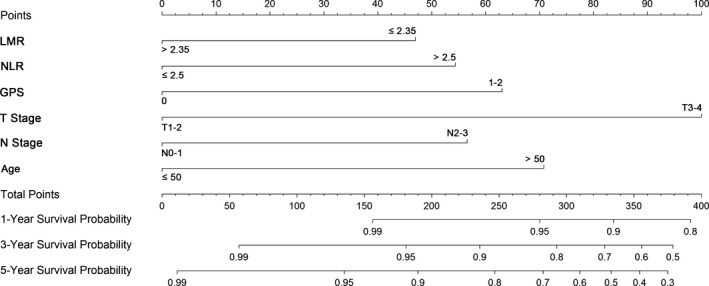
A nomogram predicts the disease‐specific survival (DSS) of patients with nasopharyngeal carcinoma. This nomogram was based on age, T stage, N stage, and inflammatory biomarkers such as the GPS, the NLR, the LMR. The total score of each patient was the sum of the points identified at the top of the scale for each factor, and was then identified on the total points scale to determine the probability of 1‐year, 3‐year, and 5‐year DSS. GPS, Glasgow prognostic score; LMR, lymphocyte‐monocyte ratio; NLR, neutrophil‐lymphocyte ratio.

To predict the prognosis for patients with NPC, a nomogram was established by involving all the independent prognostic factors above (Fig. 3). The total score of each patient was the sum of the points identified at the top of the scale for each factor, and was then identified on the total points scale to determine the probability of survival. It could predict the 1‐year, 3‐year, and 5‐year DSS of NPC patients from initial RT.

The C‐index for predicting the 3‐year DSS of the patients of the validation set was 0.734 (standard error 0.056). The calibration plot was shown in Fig. S4. Additionally, the baseline clinical characteristics of the validation set were summarized in Table S1.

## Discussion

NPC is a heterogeneous entity with wide variation in clinical progression and prognosis. Even the patients with the same TNM stage may have various outcomes. Because the TNM stage is the gold standard for predicting prognosis and guiding treatment, this discrepancy between clinical stage and prognosis may bring undertreatment or overtreatment. Prognostic factors which could improve prognosis prediction are needed. It is now known that genesis and progression of many cancers, such as lung, esophageal, colorectal, cervical, and head and neck cancers, are indeed strongly associated with long‐term inflammation 26. And anti‐inflammation agents such as aspirin have also been shown to reduce recurrence and metastases, and improve survival in some cancers like breast and colorectal cancer 27, 28. Therefore, inflammatory indexes might be choices of prognostic predictors for NPC.

Inflammatory indexes such as GPS, NLR, PLR, and LMR were proved to have the capacity of predicting survival of cancers, including NPC 13, 14, 15, 16, 17, 18, 19, 20, 21. In this analysis, we demonstrated that pretreatment GPS (HR: 2.362, 95% CI: 1.142–4.883), NLR (HR: 1.939, 95% CI: 1.004–3.761) and LMR (HR: 0.547, 95% CI: 0.298–0.996) were independent prognostic factors of NPC. This result was in accordance with the results of the previous studies. We also calculated the optimum cutoff points, which were 2.5 and 2.35 for NLR and LMR, respectively. Though patients with low PLR had a better 5‐year DSS than those with high PLR (81.9% vs. 66.1%, *P *= 0.002), PLR did not appear to be an independent prognostic factor in this analysis. Not only did we analyze the relationship between each inflammatory biomarker and clinical outcome, but we also analyzed association among these biomarkers. Through the Pearson correlation analysis, a moderate positive correlation was seen between the NLR and the PLR (*r *= 0.58, *P* < 0.001). And there was also a weak negative correlations between the PLR and the LMR (*r *= −0.213, *P *= 0.001), that is, the PLR might be influenced by these two factors.

It is known that the discrepancy between the prognosis of NPC and the TNM stage is mainly caused by the anatomy‐only basis of the TNM stage. Thus, a combination of the TNM stage and functional factors might build a new prognostic system which could classify NPC patients, especially those with the same clinical stage, into populations with different prognosis. For example, Yi et al. combined four proteins whose expression was related to inherited radioresistance of NPC with the TNM stage and developed a risk score model 29. Zeng et al. enrolled body mass index and lactate dehydrogenase into the TNM stage to build a nomogram 30. These models all showed better predicting ability than the TNM stage alone. Actually, inflammatory cells (such as neutrophils, lymphocytes, monocytes, and platelets) and proteins (such as CRP) are important functional factors because they take important parts in proliferation and metastasis of tumor cells 31. In our previous study, we developed a practical prognostic score model of NPC based on NLR and platelet count 32. The model was proved to be superior to TNM stage on predicting the 5‐year DSS of NPC patients. Besides that model, Yang et al. and Tang et al. also built prognostic models of NPC on basis of CRP 33, 34. In this analysis, we established a nomogram, by combing multiple inflammatory biomarkers (GPS, NLR, LMR) with the TNM stage, to predict the DSS of NPC patients. The prediction ability of this nomogram was validated in an independent dataset (C‐index for 3‐year DSS was 0.734). Both inflammatory cells and proteins were considered in our nomogram. This is the strength of our nomogram, compared with the previous prognostic models based on inflammatory biomarkers. Additionally, as we know, data from prospective study has no biases as retrospective data did, such as selection bias and information bias. This is another advantage of our nomogram. The result of this analysis may be of help to clinical application and popularization of the inflammatory biomarkers.

The greatest value of our nomogram is that it could be used to guide individualized treatment of NPC. Nowadays the standard management for locoregionally advanced NPC was concurrent chemoradiotherapy (CCRT) 35. However, local recurrence (5‐year recurrence rate, 13.9%) and distant metastasis (5‐year metastasis rate, 12.8%) still exists 36. Particularly, distant metastases remain the major causes of failure. More than 30% of the patients with locoregionally advanced diseases eventually died of distant failure 37. For improving the survival further, the intensity of CCT might not be effective enough. A more intensive systemic therapy such as adjuvant chemotherapy (ACT) and neoadjuvant chemotherapy (NACT) might be required. But through the results of the studies so far, a certain conclusion of the impact of ACT or NACT on locoregionally advanced NPC was still unable to make 38, 39, 40. Some oncological physicians tried to screen out the suitable patients for ACT or NACT. Hsieh used [18F]‐Fluorodeoxyglucose Positron Emission Tomography to pick out those who would benefit from ACT after CCRT 41. Du et al. also built a model to define high‐risk patients who were fit for NACT before CCRT. The predicting factors in this model included N2–3 disease, low serum albumin, thrombocytosis, and high pretreatment level of Epstein–Barr virus deoxyribonucleic acid (EBV‐DNA) 42. According to our nomogram, patients with abnormal level of the inflammatory biomarkers before RT also had a high risk for death from NPC. These patients might be the potentially appropriate population for NACT or ACT.

Indeed, several limitations should be acknowledged. First, it was a single‐institutional study with a relatively small sample size. However, the prediction ability of the nomogram, at least on 3‐year DSS, was validated in a dataset other than the one used to develop it. Second, as we discussed above, EBV‐DNA was one of the important risk factors of distant metastasis. Involvement of EBV‐DNA might improve further the prediction capacity of our nomogram. It was not included in this analysis mainly because EBV‐DNA was performed as a routine clinical test in our hospital from 2006, when the initial protocol of the 863 Program No. 2006AA02Z4B4 had been designed. Finally, not all the patients in this analysis received IMRT, which is now the routine technique of RT for NPC.

## Conclusions

In conclusion, GPS, NLR, and LMR were potential prognostic predictors for NPC. The nomogram based on these inflammatory biomarkers had an enhanced capacity of predicting DSS, and could be used to predicting prognosis and guiding treatment of patients with NPC.

## Conflict of Interest

None declared.

## Supporting information


**Figure S1.** Sponsorship certificate of the Ministry of Science and Technology of China for the 863 Program No. 2006AA02Z4B4.

**Figure S2.** ROC curves and cutoff values of NLR, PLR and LMR.

**Figure S3.** Pearson correlation analyses among NLR, PLR and LMR.

**Figure S4.** The calibration plot of 3‐year DSS prediction in the Validation Set.

**Table S1**. Baseline clinical characteristics of the development and validation sets.
