# Supplementary figures and images for: An inflammatory biomarker‐based nomogram to predict prognosis of patients with nasopharyngeal carcinoma: an analysis of a prospective study
Source: Cancer Med. 2016 Nov 10;6(1):310–9. doi: 10.1002/cam4.947 (PMC5269708; doi:10.1002/cam4.947)

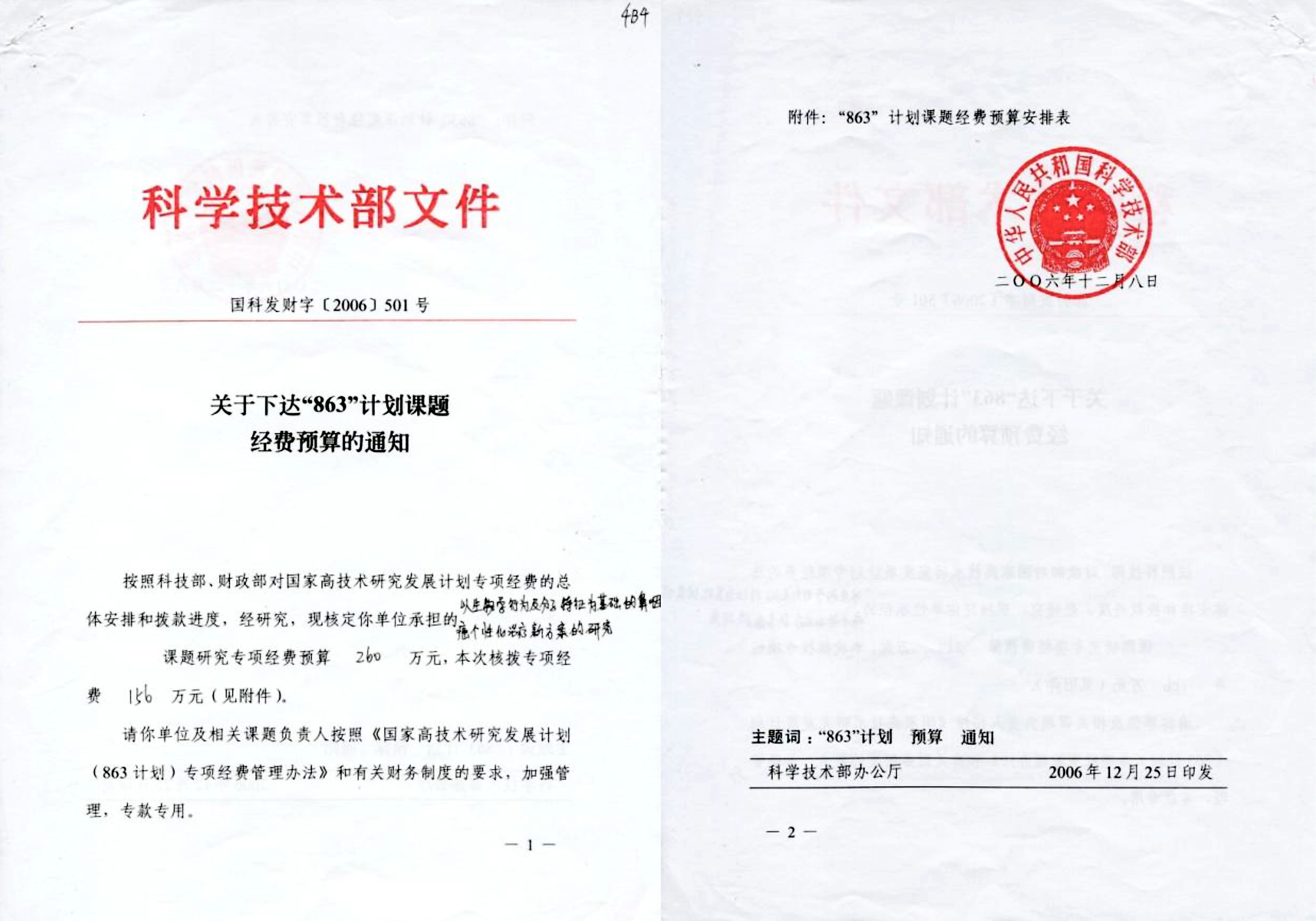

Supplement: Supplementary file 1 — Figure S1. Sponsorship certificate of the Ministry of Science and Technology of China for the 863 Program No. 2006AA02Z4B4. [file CAM4-6-310-s001.tif]

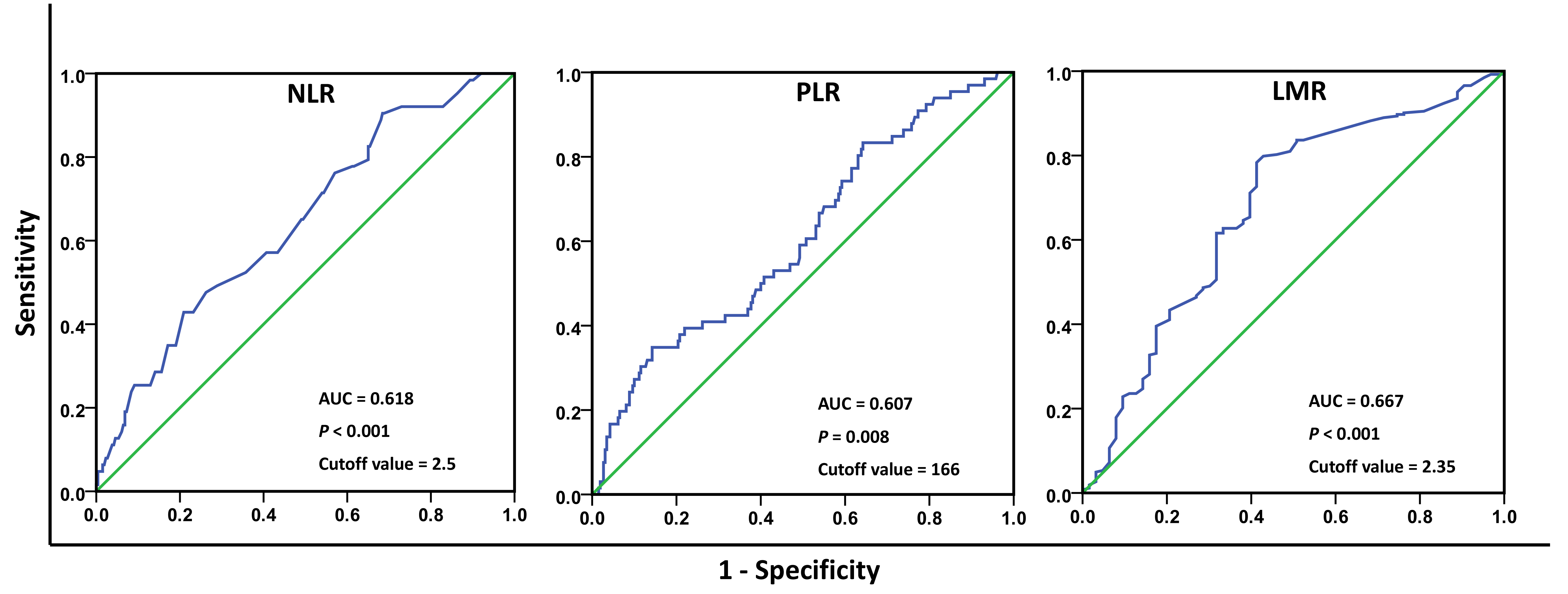

Supplement: Supplementary file 2 — Figure S2. ROC curves and cutoff values of NLR, PLR and LMR. [file CAM4-6-310-s002.tif]

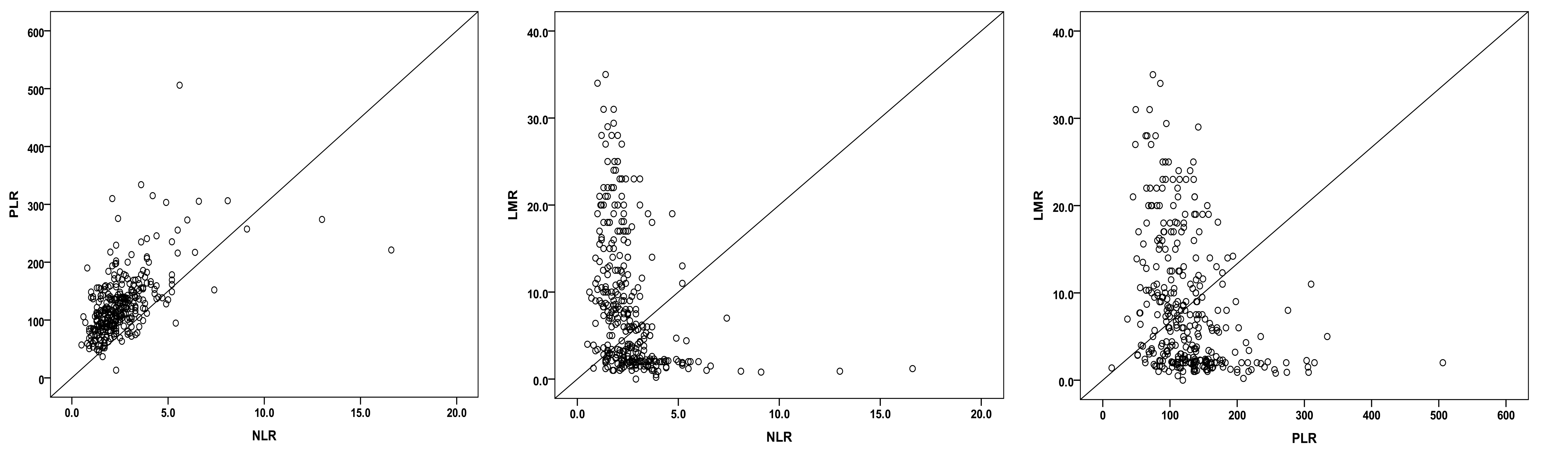

Supplement: Supplementary file 3 — Figure S3. Pearson correlation analyses among NLR, PLR and LMR. [file CAM4-6-310-s003.tif]

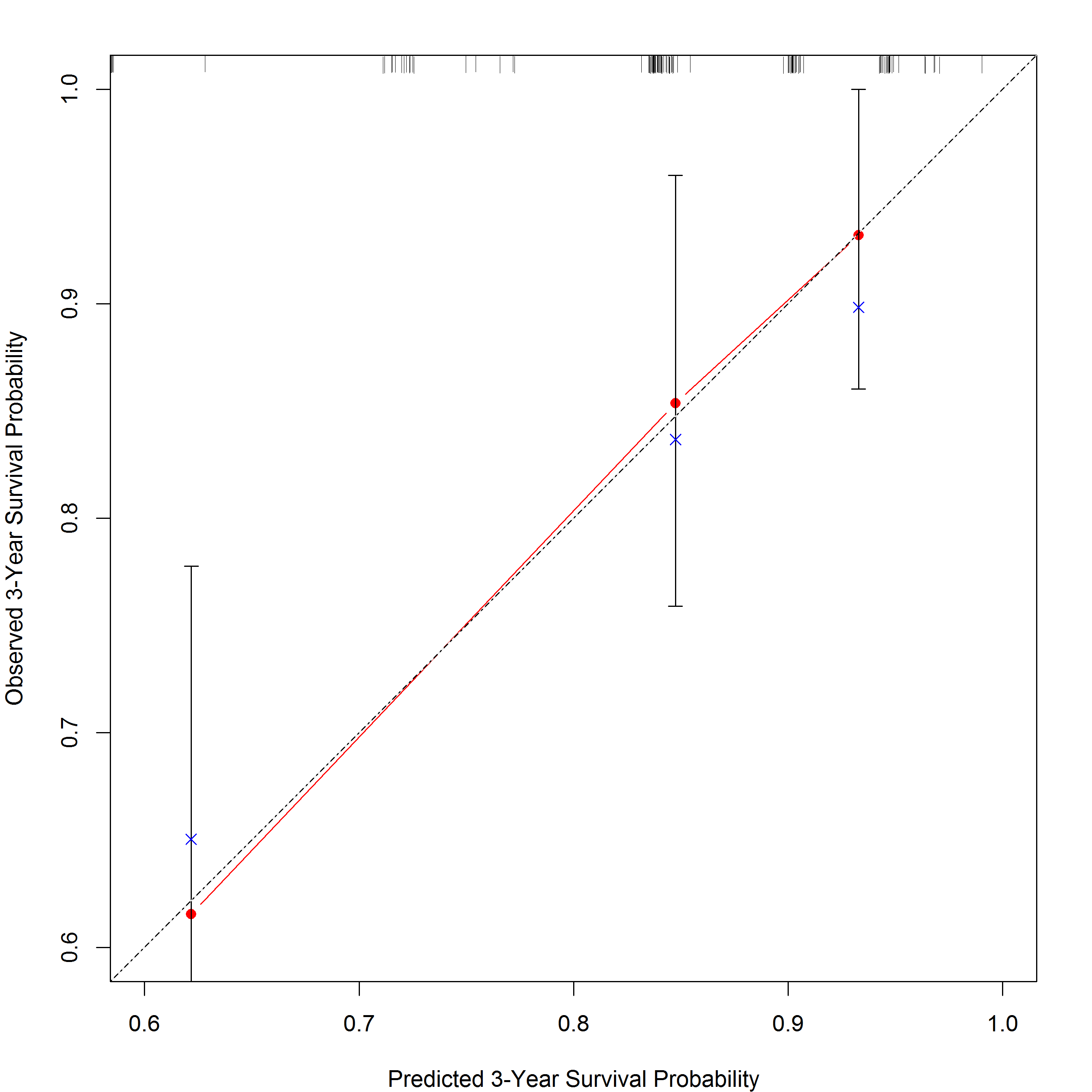

Supplement: Supplementary file 4 — Figure S4. The calibration plot of 3‐year DSS prediction in the Validation Set. [file CAM4-6-310-s004.tif]
