# Supplementary material for: An inflammatory biomarker‐based nomogram to predict prognosis of patients with nasopharyngeal carcinoma: an analysis of a prospective study
Source: Cancer Med. 2016 Nov 10;6(1):310–9. doi: 10.1002/cam4.947 (PMC5269708; doi:10.1002/cam4.947)
Supplement: Supplementary file 5 — Table S1. Baseline clinical characteristics of the development and validation sets. [file CAM4-6-310-s005.docx]

**Table S1 Baseline clinical characteristics of the Development and Validation Sets.**

| **Characteristic** | **Development**  **Set** | **Validation**  **Set** | **Χ^2^** | ***P* Value** |
| --- | --- | --- | --- | --- |
| Gender |  |  |  |  |
| Male | 184 | 98 | 0.517 | 0.472 |
| Female | 65 | 41 |  |  |
| Age |  |  |  |  |
| ≤ 50 | 164 | 97 | 0.623 | 0.430 |
| > 50 | 85 | 42 |  |  |
| KPS |  |  |  |  |
| ≤ 80 | 54 | 20 | 3.078 | 0.079 |
| > 80 | 195 | 119 |  |  |
| Clinical stage |  |  |  |  |
| I-II | 32 | 25 | 1.876 | 0.171 |
| III-IV | 217 | 114 |  |  |
| T stage |  |  |  |  |
| T1-2 | 65 | 49 | 3.597 | 0.058 |
| T3-4 | 184 | 90 |  |  |
| N stage |  |  |  |  |
| N0-1 | 118 | 69 | 0.181 | 0.671 |
| N2-3 | 131 | 70 |  |  |
| IMRT |  |  |  |  |
| No | 180 | 30 | 92.37 | < 0.001 |
| Yes | 69 | 109 |  |  |
| Undifferentiated  carcinoma |  |  |  |  |
| No | 25 | 14 | 0.000 | 0.992 |
| Yes | 224 | 125 |  |  |
| CCT |  |  |  |  |
| No | 13 | 7 | 0.006 | 0.937 |
| Yes | 236 | 132 |  |  |

KPS, Karnofsky performance score; IMRT, intensity-modulated radiotherapy; CCT, concurrent chemotherapy.
